# Supplementary material for: Shape‐Guided Mixed Metro Map Layout
Source: Comput Graph Forum. 2023 Mar 20;41(7):495–506. doi: 10.1111/cgf.14695 (PMC10946624; doi:10.1111/cgf.14695)
Supplement: Supplementary file 1 — Supporting Information [file CGF-41-495-s001.pdf]

Appendix A: Path Invariant Testing Experiment

In this section, we would like to show several examples to demonstrate the proposed approach is translation and scale invariant, as explained in Section 6. For each test case, we generate an arbitrary guide shape with random size and position. The results are shown in Figure 16, where we show the position and scale of the guide shape in the input image and the resulting path in the corresponding output image. Figures 16(a)-(d) show the examples of Taipei metro system, Figures 16(e)-(f) are the examples of Montreal metro system, and Figures 16(g)-(h) depict the examples of Paris metro system. The guide shapes include a heart, a flower, and an eye, respectively. Based on this experiment, shape size and position are invariant since the found paths are identical even though the shape size and position are different.

© 2022 The Author(s)
Computer Graphics Forum © 2022 The Eurographics Association and John Wiley & Sons Ltd.

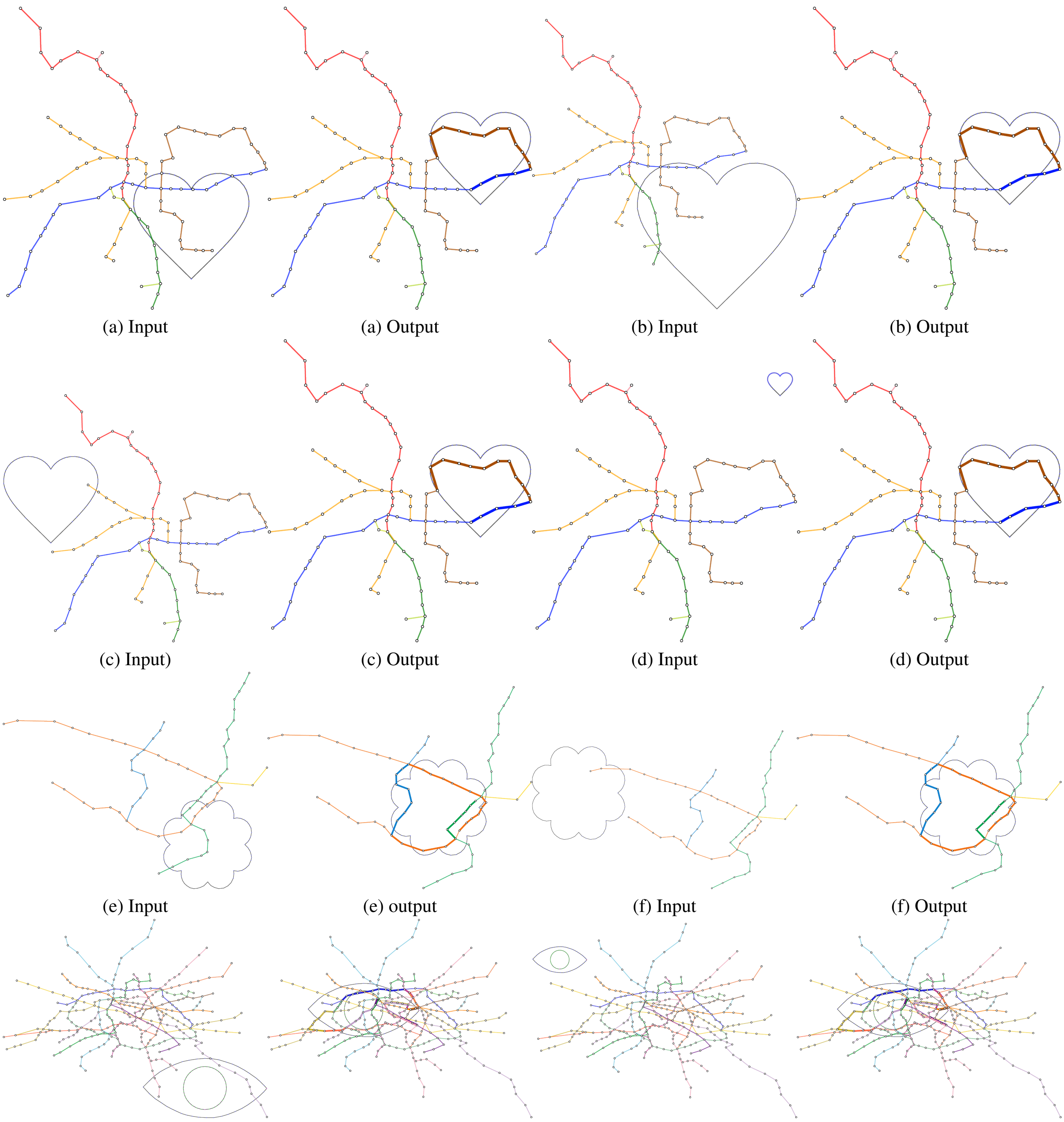

(a) Input (a) Output (b) Input (b) Output

(c) Input) (c) Output (d) Input (d) Output

(e) Input (e) output (f) Input (f) Output

(g) Input (g) Output (h) Input (h) Output

Figure 16: *Results of the translation and scale invariant test. For each test-case, we show the input guide shape (gray) in the input image and the resulting path (highlighted with a bold line) in the output image.*

© 2022 The Author(s)
Computer Graphics Forum © 2022 The Eurographics Association and John Wiley & Sons Ltd.

Appendix B: Parameter Testing Experiment

To visually show the effects of different weight values in Equations (1) and (7), we selected different parameter values for demonstration (see Figures 17-20). In Figure 17(a) and 19(a), we applied the weights $w_c = 4$, $w_l = 1$, $w_a = 2$, and $w_p = 0.16$ for the smooth process. For the test-case 1, we adapted $w_c = 10.0$, for test-case 2, we used $w_a = 0.5$, and for test-case 3, we set $w_a = 5.0$. The corresponding results are shown in Figures 17(b)-(d) and 19(b)-(d). For the mixed layout, we applied the default weights $w_o = 2.0$, $w_p = 0.1$, and $w_c = 10.0$. The default test-case and the test cases 1 to 3 are computed with these default mixed weights.

The effects of weights for the mixed process are depicted in Figures 18 and 20. For the test-cases 4 to 7, the smooth layout is computed with the default parameters, and for the test case 4, we adapted w_o and w_c to 5.0. For the test-case 6, we use $w_o = 10.0$ and for the test-case 7, we applied $w_c = 2$. The results of the effects are further summarized in the following paragraphs.

Smooth Layout Variables (Test-cases 1-3)

In the smooth results of Taipei with the default parameters (Figure 17(a)), one can see that the metro lines are straightened and stations are evenly spaced apart. Test-case 2 depicts the effect of w_a clearly (Figure 17(c) and 19(c)). By decreasing the w_a, the system prioritizes the other objective functions and metro lines are less straight, resulting in a less clear layout after the mixed stage. By increasing w_a (Test-case 3, Figure 17(d) and 19(d)) metro lines are even more straightened at the cost of the other objective functions. This results in a layout that does not preserve the geographic shape of the metro network. For a more complex network like Moscow, the effects of the different parameters are even more significant. This indicates that the potential conflicts between the different objective functions and that the system is not able to find a reasonable solution. For example, if w_a is decreased, the approach does not produce appealing results.

Mixed Layout Variables (Test-cases 4-7)

For the Taipei test data, the variation of the weights w_o and w_c in the mixed stage have relatively little effect on the results of the final layout, as one can see in Figure 18. This implies that the process is relatively stable and the system can find a good solution for the objective function. For the Moscow test cases, the system fails to rotate a large amount of edges to octolinear direction, independently from the parameters, especially in the dense center of the map. Therefore, when the weight w_c is decreased (e.g., Test-case 5 shown in Figure 20(e)), the system prioritizes the octolinear edge constraint and pushes stations away from the guide that should be located close to the guide shape.

When comparing different results of the smaller and less complex metro systems to the larger and more complex Moscow system, we can see that in the complex systems, the weights have to be balanced carefully, while in simple networks the system produces good potential results for a variety of parameters and finds a reasonable solution that satisfies different objective terms.

© 2022 The Author(s)
Computer Graphics Forum © 2022 The Eurographics Association and John Wiley & Sons Ltd.

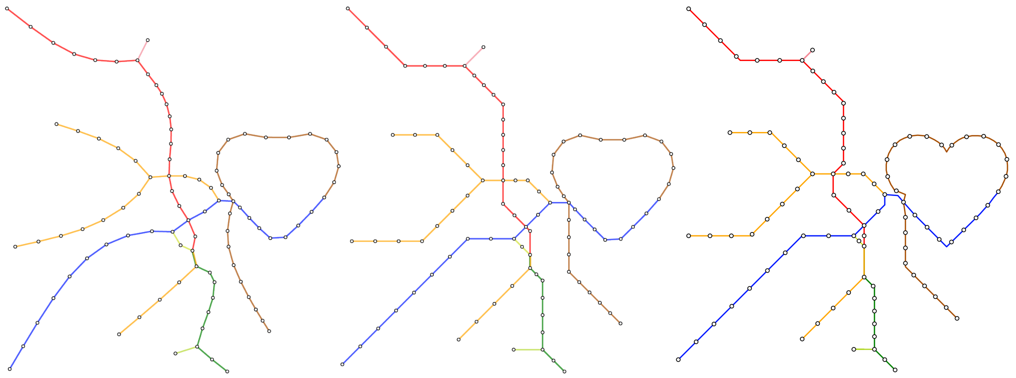

(a) Default Test-case; Parameters for the smooth stage: $w_c = 4$, $w_l = 1$, $w_a = 2$, $w_p = 0.16$.

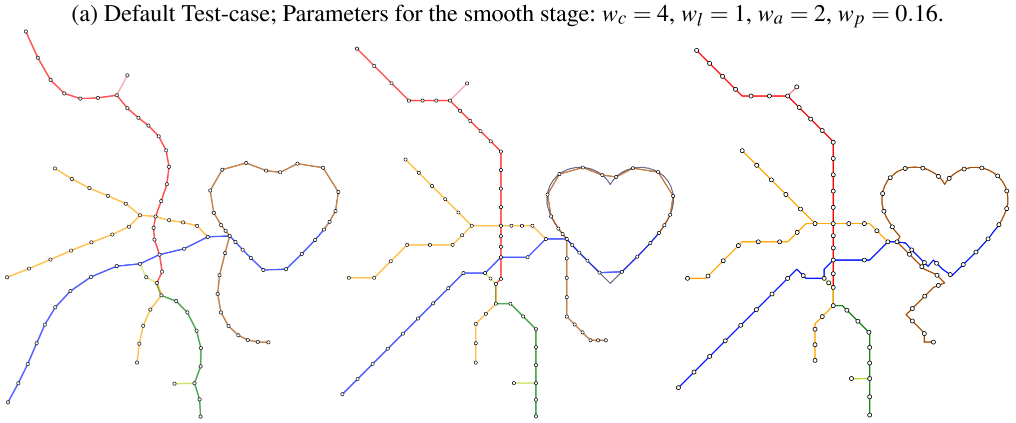

(b) Test-case 1; Parameters for the smooth stage: $w_c = 10$, $w_l = 1$, $w_a = 2$, $w_p = 0.16$.

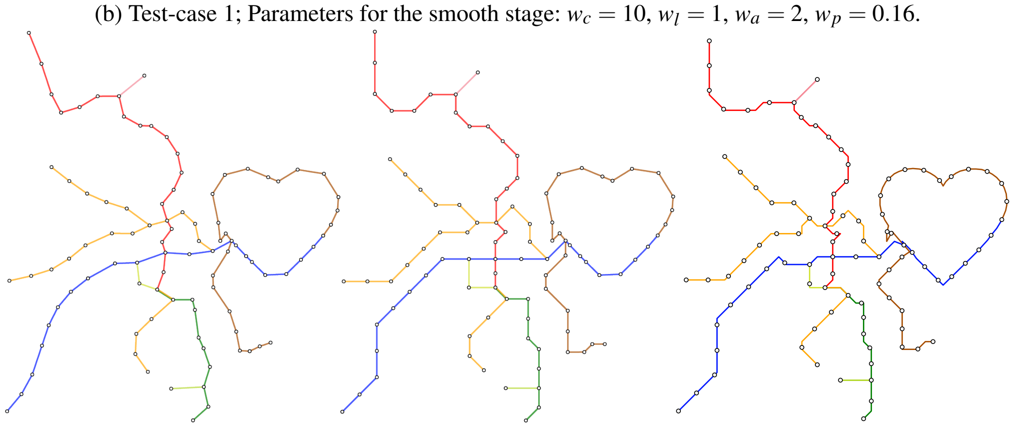

(c) Test-case 2; Parameters for the smooth stage: $w_c = 4$, $w_l = 1$, $w_a = 0.5$, $w_p = 0.16$.

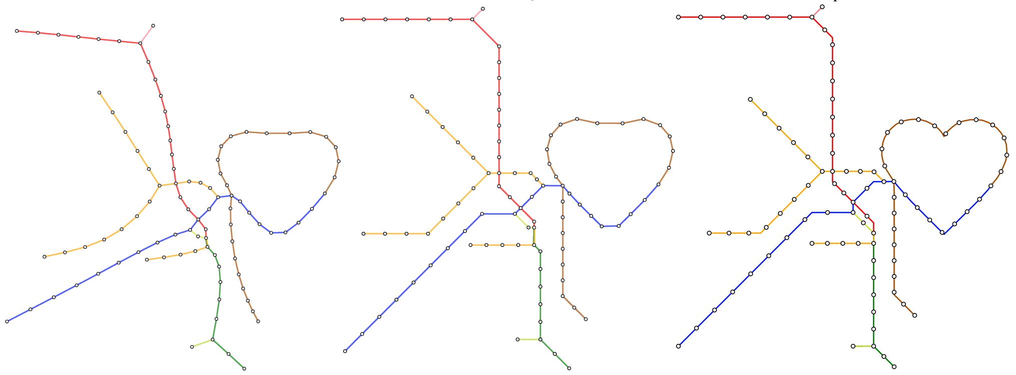

(d) Test-case 3; Parameters for the smooth stage: $w_c = 4$, $w_l = 1$, $w_a = 5.0$, $w_p = 0.16$.

Figure 17: *Taipei metro system computed with different weights for the smooth optimization process. The mixed stage was computed with the parameters* $w_o = 2$*,* $w_p = 0.1$ *and* $w_c = 10$*. Left: smooth layout, center: mixed layout and right: grid aligned layout.*

© 2022 The Author(s)
Computer Graphics Forum © 2022 The Eurographics Association and John Wiley & Sons Ltd.

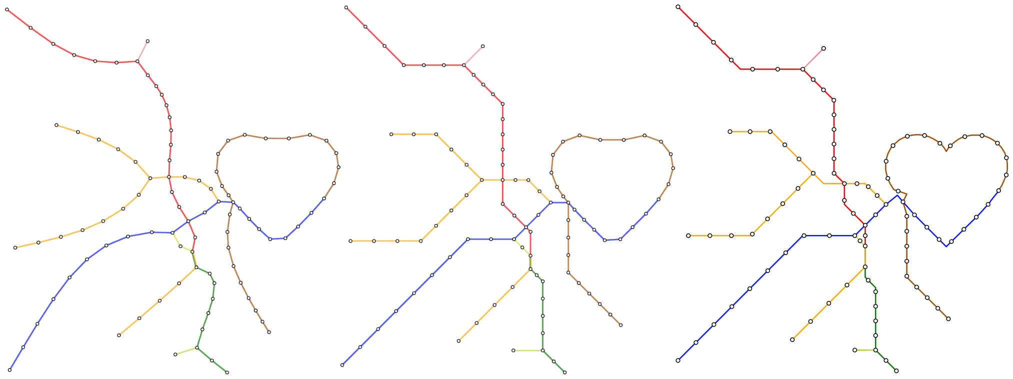

(e) Test-case 4; Parameters for the mixed stage: $w_o = 5$, $w_p = 0.1$, $w_c = 5$.

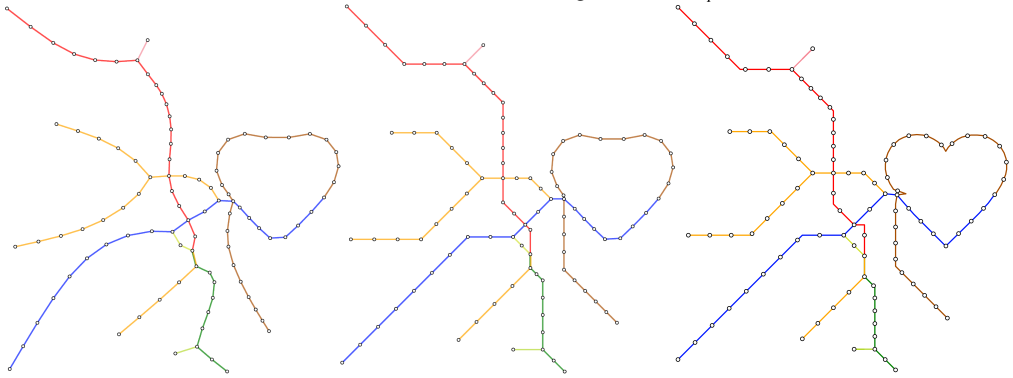

(f) Test-case 5; Parameters for the mixed stage: $w_o = 10$, $w_p = 0.1$, $w_c = 2$.

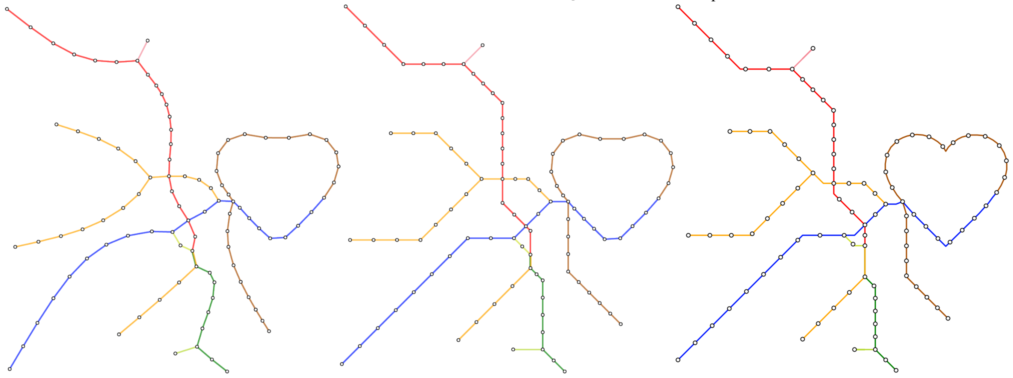

(g) Test-case 6; Parameters for the mixed stage: $w_o = 10$, $w_p = 0.1$, $w_c = 10$.

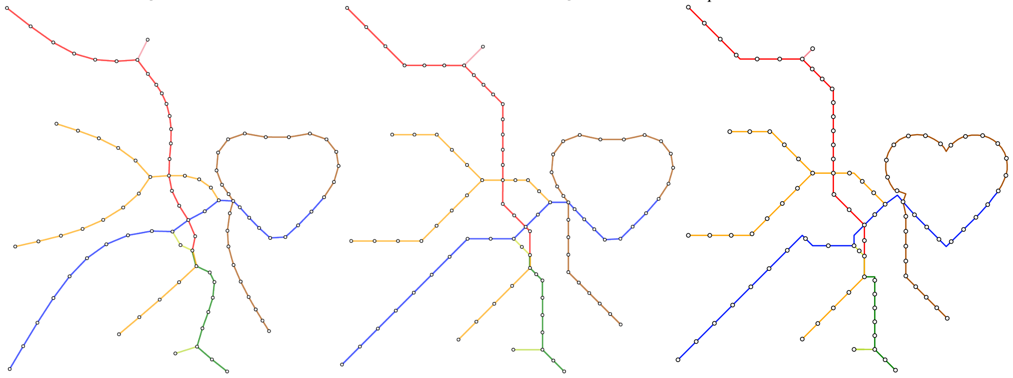

(h) Test-case 7; Parameters for the mixed stage: $w_o = 2$, $w_p = 0.1$, $w_c = 2$.

Figure 18: *Taipei metro system computed with different weights for the mixed optimization process. The smooth stage was computed with the parameters $w_c = 10$, $w_l = 1$, $w_a = 2$ and $w_p = 0.16$. Left: smooth layout, center: mixed layout and right: grid aligned layout.*

© 2022 The Author(s)
Computer Graphics Forum © 2022 The Eurographics Association and John Wiley & Sons Ltd.

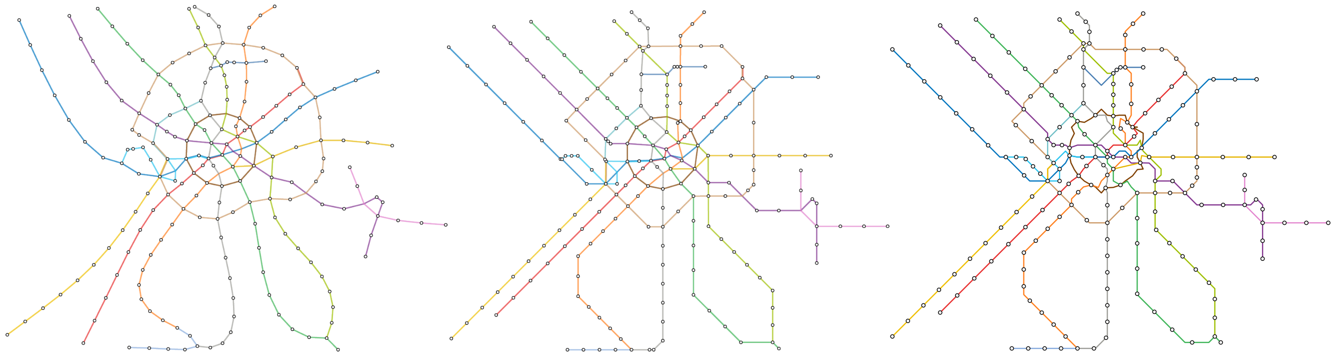

(a) Default Test-case; Parameters for the smooth stage: $w_c = 4$, $w_l = 1$, $w_a = 2$, $w_p = 0.16$.

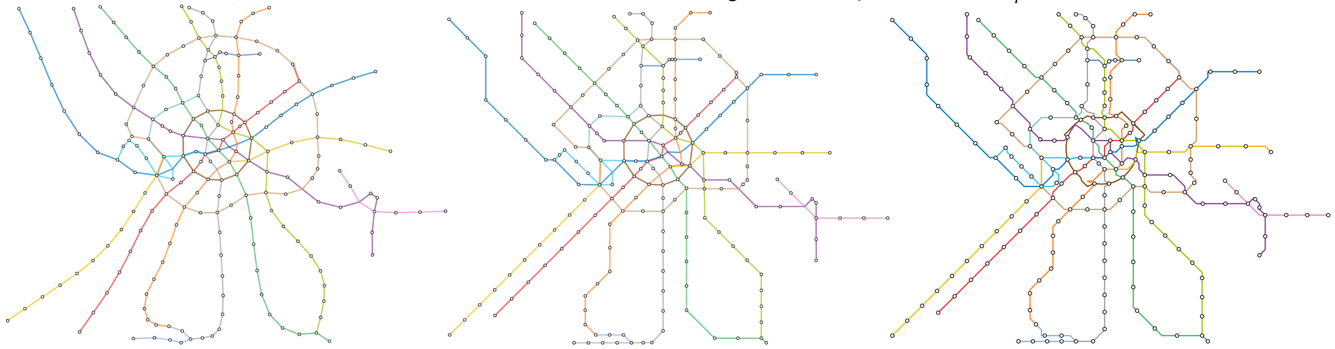

(b) Test-case 1; Parameters for the smooth stage: $w_c = 10$, $w_l = 1$, $w_a = 2$, $w_p = 0.16$.

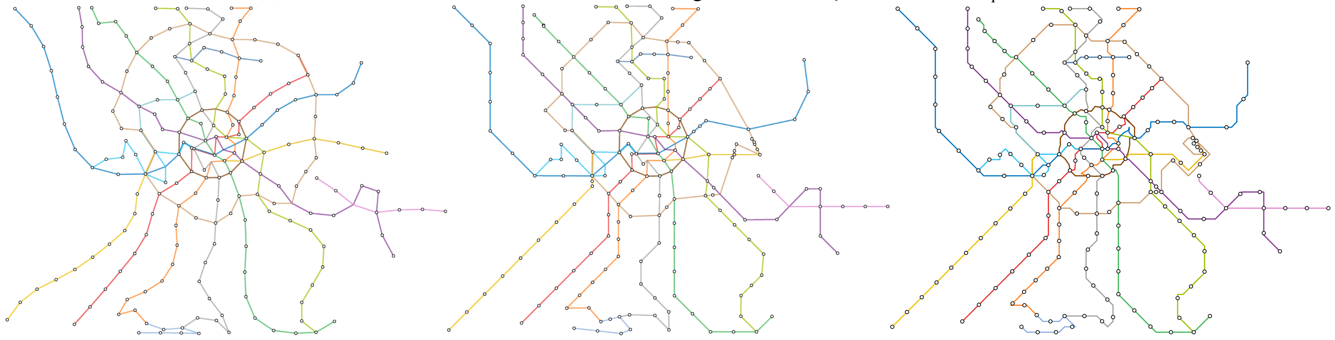

(c) Test-case 2; Parameters for the smooth stage: $w_c = 4$, $w_l = 1$, $w_a = 0.5$, $w_p = 0.16$.

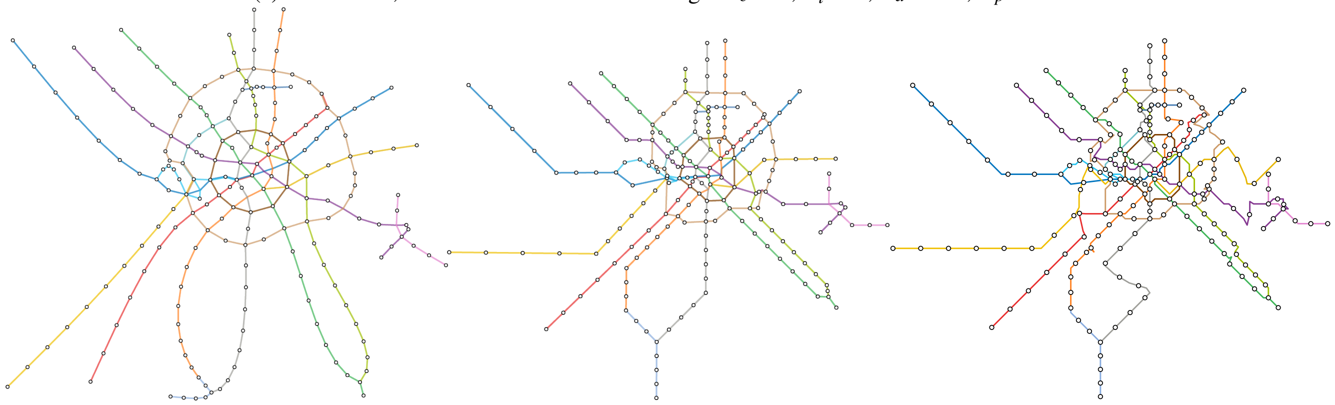

(d) Test-case 3; Parameters for the smooth stage: $w_c = 4$, $w_l = 1$, $w_a = 5.0$, $w_p = 0.16$.

Figure 19: *Moscow metro system computed with different weights for the smooth optimization process. The mixed stage was computed with the parameters* $w_o = 2$, $w_p = 0.1$ *and* $w_c = 10$. *Left: smooth layout, center: mixed layout and right: grid aligned layout.*

© 2022 The Author(s)
Computer Graphics Forum © 2022 The Eurographics Association and John Wiley & Sons Ltd.

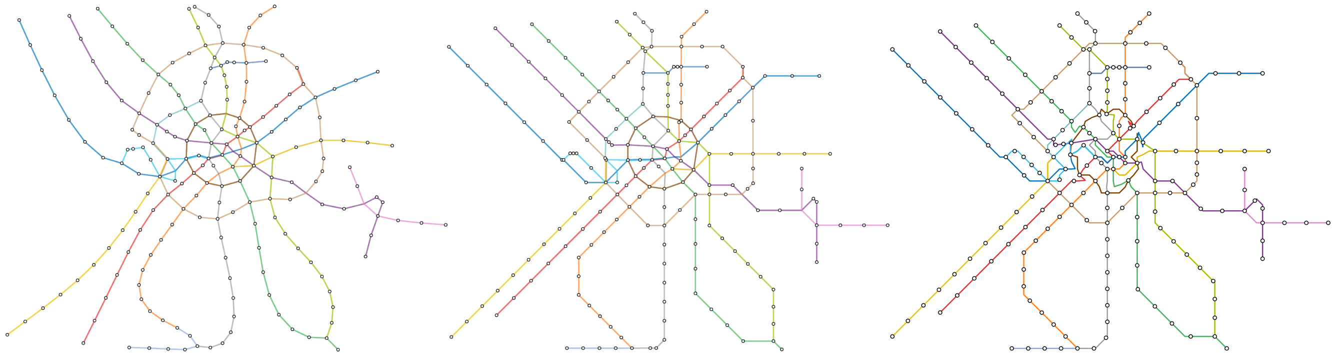

(e) Test-case 4; Parameters for the mixed stage: $w_o = 5$, $w_p = 0.1$, $w_c = 5$.

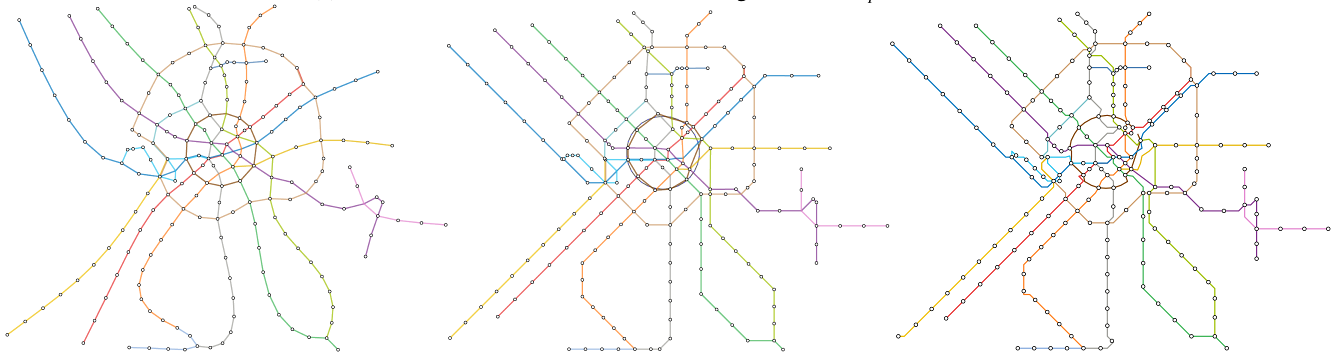

(f) Test-case 5; Parameters for the mixed stage: $w_o = 10$, $w_p = 0.1$, $w_c = 2$.

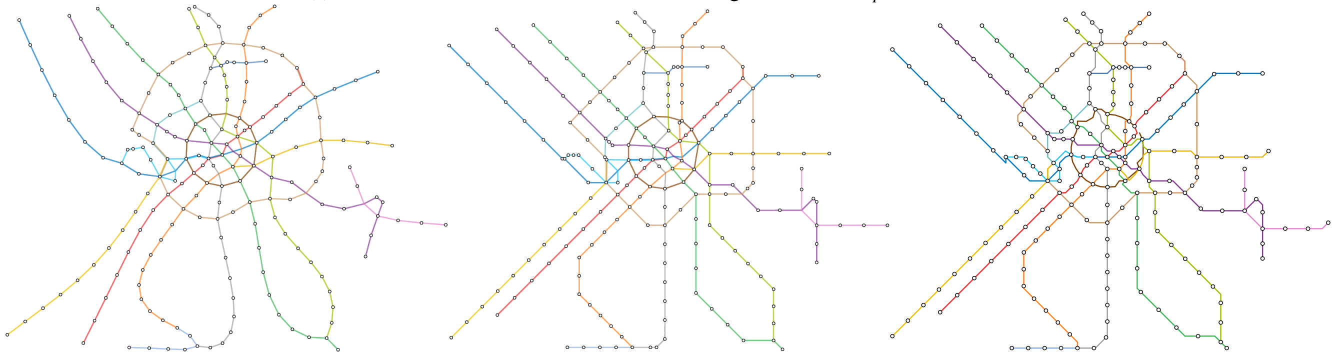

(g) Test-case 6; Parameters for the mixed stage: $w_o = 10$, $w_p = 0.1$, $w_c = 10$.

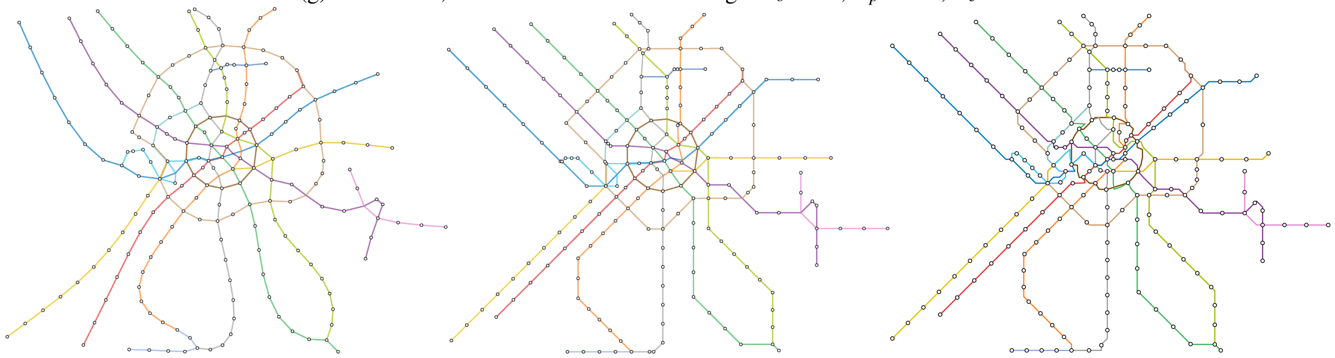

(h) Test-case 7; Parameters for the mixed stage: $w_o = 2$, $w_p = 0.1$, $w_c = 2$.

Figure 20: *Moscow metro system computed with different weights for the mixed optimization process. The smooth stage was computed with the parameters $w_c = 10$, $w_l = 1$, $w_a = 2$ and $w_p = 0.16$. Left: smooth layout, center: mixed layout and right: grid aligned layout. Note that grid alignment layouts can have up to 5 edges, which could not be routed.*

© 2022 The Author(s)
Computer Graphics Forum © 2022 The Eurographics Association and John Wiley & Sons Ltd.
